# 

**Published:** 1900-01-13

**Authors:** 


					THE HOSPITAL. "The standard of highest purity."?Lancet. January 13th, 1900.
OADJ^.RYS f BE CADo^RYS
? .....    W/ Nfl nOTiriB no at vat tpo ffoon
ABSOLUTELY PURE. ^ **? DRUGS OR ALKALIES USED.
rutAscoir Western InfYrmar
No. 694. I Yol. XXVII. [JSSSSiH Saturday,
Jan. 13, 1900.
[Subscription Terms,J pRICE TWOPENCE.

				

